# Inorganic Membranes: Preparation and Application for Water Treatment and Desalination

**DOI:** 10.3390/ma11010074

**Published:** 2018-01-05

**Authors:** Ahmad Kayvani Fard, Gordon McKay, Anita Buekenhoudt, Huda Al Sulaiti, Filip Motmans, Marwan Khraisheh, Muataz Atieh

**Affiliations:** 1 Qatar Environment and Energy Research Institute, Hamad Bin Khalifa University, Qatar Foundation, Doha 5825, Qatar; afard@qf.org.qa (A.K.F.); halsulaiti@hbku.edu.qa (H.A.S.); mkhraisheh@hbku.edu.qa (M.K.); 2 College of Science and Engineering, Hamad Bin Khalifa University, Qatar Foundation, Doha 5825, Qatar; gmckay@qf.org.qa; 3 Department of Separation and Conversion Technology, VITO (Flemish Institute of Technological Research), Boeretang 200, B-2400 Mol, Belgium; anita.buekenhoudt@vito.be (A.B.); filip.motmans@vito.be (F.M.)

**Keywords:** inorganic membrane, ceramic, water, wastewater, zeolite, silica, hybrid membrane, mixed matrix

## Abstract

Inorganic membrane science and technology is an attractive field of membrane separation technology, which has been dominated by polymer membranes. Recently, the inorganic membrane has been undergoing rapid development and innovation. Inorganic membranes have the advantage of resisting harsh chemical cleaning, high temperature and wear resistance, high chemical stability, long lifetime, and autoclavable. All of these outstanding properties made inorganic membranes good candidates to be used for water treatment and desalination applications. This paper is a state of the art review on the synthesis, development, and application of different inorganic membranes for water and wastewater treatment. The inorganic membranes reviewed in this paper include liquid membranes, dynamic membranes, various ceramic membranes, carbon based membranes, silica membranes, and zeolite membranes. A brief description of the different synthesis routes for the development of inorganic membranes for application in water industry is given and each synthesis rout is critically reviewed and compared. Thereafter, the recent studies on different application of inorganic membrane and their properties for water treatment and desalination in literature are critically summarized. It was reported that inorganic membranes despite their high synthesis cost, showed very promising results with high flux, full salt rejection, and very low or no fouling.

## 1. Introduction

Today, membrane-based separation processes are widely used in our day-to-day activities from the petrochemical, food, biotechnology and pharmaceutical industries, and in a range of environmental uses, including water treatment and desalination. Their simplicity and cost efficiency compared to other conventional separation processes such as adsorption, absorption, or distillation have made them very popular and wide spread in their applications [1].

Low energy consumption, ease of scaling up, ability to hybridize with other processes, continuous operation, high intensity and automatic operation are among the main advantages of membrane processes. On the other hand, their limitations include the membrane fouling, limited chemical stability and short lifetime. Intensive R&D has been undertaken to improve the membrane properties. An efficient membrane should provide reliable use, produce a high flux at lower pressure, require less footprint, provide higher water quality, and have less pretreatment requirements [1].

With the advancement in materials science and technology developments, membranes have now taken a significant share of the separation industry. Recent financial reports show that the global demand for membranes and membrane modules reached 15.6 billion USD and is expected to grow annually by 8% in the future [2]. Most of the membranes’ market share is dedicated to polymeric membranes due to their lower cost. However, polymeric membranes suffer from low mechanical stability and fouling problems. On the other hand, inorganic membranes possess better properties such as high chemical, thermal and mechanical stabilities, which make them suitable for use in harsh conditions such as corrosive and high temperature environments [3,4,5]. 

**Figure 1:**
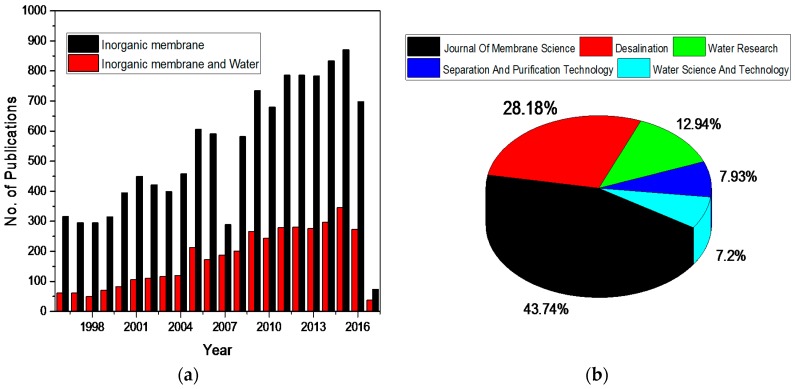
(**a**) The total number of publications on inorganic membranes in the literature according to Scopus database and (**b**) distribution of papers in the corresponding journals.

Different types of inorganic membranes have been developed according to the literature. As this area attracts the attention of the researcher worldwide, the number of annual publications returned by the Scopus database, when “inorganic membranes” was used as the keyword, is continuously growing from less than 450 publications in 2000 to double in 2016 as shown in Figure 1a. In addition to that, the publications in the application of inorganic membranes for water treatment and desalination increased from less than 21% in 2000 to about 40% in 2016. It is also worth noting that 40% of publications on the applications of inorganic membranes in water treatment and desalination appear in 5 journals, i.e., Journal of Membrane Science (43%), Desalination (28%), Water Research (12%), Separation and Purification Technology (8) and Water Science and Technology (7.2%). 

Although many review articles and book chapters have been published previously on specific types of inorganic membranes such as zeolite membranes [6,7], ceramic membranes [8,9], modeling of inorganic membranes [10], and gas separation using inorganic membranes [11] but a comprehensive and timely review on the different types of inorganic membranes for water treatment and desalination applications is not available in the literature. 

In this review article, a critical evaluation of the recent advances in the development of various inorganic membranes for water treatment applications and desalination are provided. This review should help researchers to have a better understanding of the fundamental aspects of separation by different types of inorganic membranes. This review contains a short description of the basic principles of membrane processes, followed by an analysis of recent studies on the development of advanced inorganic membranes in water treatment. Subsequently, hybrid organic and inorganic membranes are also reviewed and compared. Finally, conclusions and future perspectives regarding the development of inorganic membranes for water treatment applications are provided.

## 2. Membranes’ Matrix

The membrane matrix and materials used for its synthesis are vital factors that control the membrane performance in the separation process. The type of material defines the separation mechanism as well as the morphology of the fabricated membrane. Material selection for membrane preparation is not a random practice but is based on the defined properties of a selected material originating from its structural features and chemical and physical nature. The factors that play a role in the material selection can be: (1) selectivity and permeability of the material; (2) chemical resistivity; (3) mechanical robustness; (4) thermal resistance; and (5) economical and engineering feasibility [12,13].

**Figure 2:**
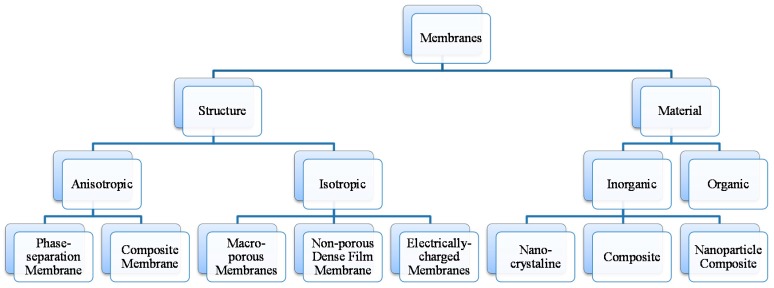
Membrane categories based on their structure and material.

In general, membranes are categorized into two broad classes based on their cross section and based on the material used for their synthesis as shown in Figure 2. 

For the first class, membranes are divided into two groups, namely, isotropic and anisotropic membranes. Isotropic membranes have a homogenous composition where their structure is made up of a single material. Isotropic membranes can be sub-classified into macroporous membranes, nonporous dense films, and electrically charged membranes. Macroorous membranes, also called sieving membranes, are those which filter out solutes based on their size and the size of the membranes pores. Their pore diameter may vary from 0.1–5 μm [14,15]. Nonporous dense film membranes are those membranes where the transport of solute occurs by applying a force such as pressure, concentration, or electric-field gradients. Therefore, these types of membranes are diffusion driven based on the concentration difference on each side of the membrane and the solute transfer is governed by the transport rate [16]. Electrically charged membranes or anion/cation exchange membranes are those membranes whose surfaces have been enhanced by negative or positive ions. They are made of either nonporous dense films or microporous structures. The transport mechanism in such membranes is governed by the ion concentration and charge density of the solute [17]. 

On the other hand, anisotropic membranes have a heterogeneous composition both in their chemical composition as well as in their structure. Anisotropic membranes are further divided into phase-separation membranes (Loeb-Sourirajan membranes) and composite membranes such as thin-film, coated films and self-assembled structures. Phase separation membranes have a homogenous chemical composition but their structure, pore size, porosity and thickness across the membranes vary from one point to another [18]. Instead, composite membranes like thin-film membranes have a heterogeneous chemical composition and structure. Thin film membranes consist of a thin layer on top and a thick porous support made of polymeric material. The transport of solute across thin film membranes is predominantly governed by the thin surface layer and its thickness, porosity, pore size, etc. [19]. 

Membranes can be categorized based on the material of synthesis and they are divided into organic (polymeric) and inorganic membranes. Organic membranes are those made of polysulfone, polyethersulfone, cellulose acetate, polymethylpentene, polyimide, polyetherimide, polycarbonate, polydimethylsiloxane; and polyphenyleneoxide while inorganic membranes include ceramics, carbon molecular sieves, nanoporous carbon, mixed conducting perovskites, zeolites, amorphous silica, and palladium alloys [20].

Molecular sieve membranes are more chemically resistant to organic solvents, chlorine and other chemicals compared to organic membranes. This is very helpful for water treatment applications where water is dosed with chlorine or other disinfectants which may degrade organic membranes. Chemical resistivity also allows inorganic membranes to be cleaned and washed regularly with different solvents and anti-scaling compounds. Additionally, inorganic membranes are not vulnerable to microbial attack and they are mechanically robust. 

Currently, polymeric membranes are used more frequently especially in water treatment and desalination regardless of their drawbacks, such as stability at elevated temperatures and fouling. Therefore, significant R&D efforts are being undertaken nowadays for the development of inorganic membranes. Although inorganic membranes are more expensive compared to polymeric membranes, they have advantages such as withstanding harsh chemical cleaning and frequent backwashing, the ability to be sterilized and autoclaved, high temperature resistance ( up to 500 °C) and wear resistance, well-defined and stable pore structure, high chemical stability, and a long life time. Nevertheless, their high cost and stiffness are their main drawbacks [11,21]. Table 1 provides a comparison between organic and inorganic membranes in terms of their material characteristics, advantages and disadvantages.

Generally speaking, for water purification applications, it is essential for the membrane to be resistant to the exposed separation environments. Also, due to the applied pressure difference as the driving force, the membrane should maintain good mechanical rigidity and stability against the applied stress. Feed streams are usually contaminated with other chemical components such as organics (aromatic compounds, oils and solvents) that may swell or dissolve the membrane materials, so the membrane materials have to be chemically resistant to the environment to increase their lifetime. Temperature stability is also an important factor for the membrane to tolerate high feed operating temperatures and avoid damage. Since the last decade, membrane material technology has been developing very rapidly. Many researches have been undertaken to investigate various types of material and their structure to be used for a wide variety of applications. Membrane synthesis, based on the material used, is categorized mainly into organic (polymeric) and inorganic membranes. Organic membranes are those made of nonporous polymeric material and inorganic membranes are those containing metals, oxides, or elementary carbon in their structure [27]. Examples of organic membranes are polysulfone, polyethersulfone, cellulose acetate, polymethylpentene, polyimide, polyetherimide, polycarbonate, polydimethylsiloxane, and polyphenyleneoxide whereas examples of inorganic membranes can include carbon molecular sieves, nanoporous carbon, mixed conducting perovskites, zeolites, amorphous silica, and palladium alloys [20].

Advantages of organic membranes are cost-effectiveness, good selectivity and easy processability. This type of membrane holds the largest share of the membrane separation technology market with activity on both an industrial scale applications and in academic research. Amorphous polymers can exist in both glassy or rubbery states based on their operating temperatures. Polymers at their glass transition state are hard and rigid, but they become soft and flexible when transferred to rubbery state. The temperature at which they transfer from the glassy state to the rubbery state is denoted as the glass transition temperature, Tg, and defined as the temperature at which the thermal expansion coefficient changes in going from the rubbery state to the glassy state [28]. 

For water and gas separations, rubbery membranes show higher fluxes compared to glassy membranes. However, the separation efficiencies for rubbery materials are lower due to their small diffusivity selectivity. Consequently, glassy polymers are naturally more size and shape selective, and as a result they are more selective and mechanically more stable when compared to the rubbery materials. Therefore, the glassy polymers caught more interest in industry and they are mostly used for membrane separation systems around the globe [29]. Most of the membranes used for water and gas separations are polycarbonates, polysulfones, polyesters, polypyrrolones and polyimides [13]. 

On the other hand, the market for inorganic membranes still small and it is anticipated to increase in the near future. However, global attention towards inorganic membranes is rapidly growing. Inorganic membranes, especially silica, porous glass, crystalline zeolites, microporous beryllium oxide powders and carbon membranes created a lot of attention due to their capability in having both higher permeability and selectivity [30,31,32]. This phenomena is called the “upper bound trade-off curve” and was first introduced by Robeson [23]. This phenomenon describes the inverse relationship between the permeability and selectivity for the gas separation performance of various membrane materials [23]. For instance, when the gas selectivity increases, the gas permeability decreases and vice versa. For this reason, inorganic membranes have a superior performance over glassy polymeric membranes. The first inorganic membrane was introduced as a molecular sieving material (MSM) by J.W. McBain in the 1930s where it was acting as a sieve on a molecular scale [33]. The material showed a very interesting performance with good productivity and selectivity [33]. Inorganic membranes can also operate at higher temperatures compared to organic membranes for their application to gas separation processes where the temperature can go up to 300 °C. Molecular sieve membranes are chemically resistant to organic solvents, chlorine and other chemicals and more resistant than organic membranes. This property can be very helpful for water treatment applications where water is dosed with chlorine and/or other disinfectants and organic membranes could fail. Also chemical resistivity allows inorganic membranes to be cleaned and washed regularly with different solvents and anti-scaling agents. Additionally, inorganic membranes are not vulnerable to microbial attack, and they are mechanically robust. 

Since this review is concerned with the techniques to synthesize inorganic membranes and their applications in the separation and membrane industry, then the development of inorganic membranes is first discussed, followed by the different techniques for inorganic membrane modification and fabrication. Finally, recent and novel studies on the preparation of these materials are discussed and critically summarized.

## 3. Structure of Inorganic Membranes

**Figure 3:**
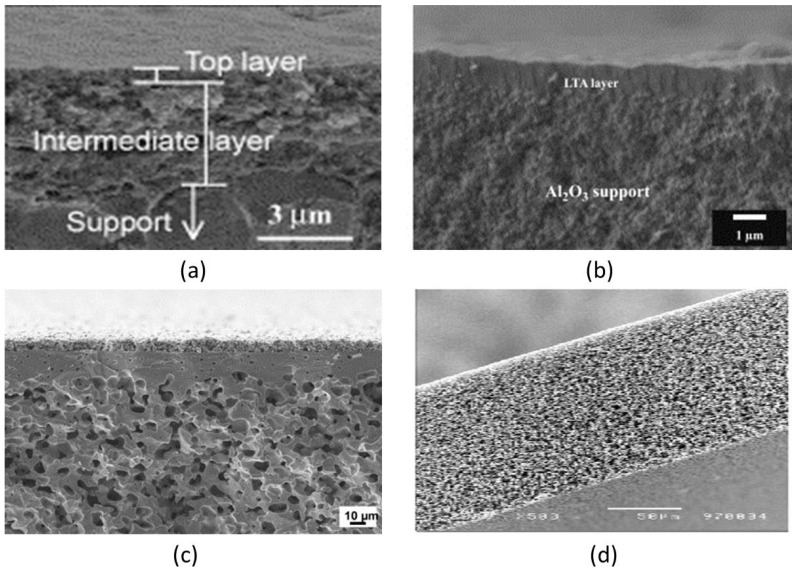
SEM image of (**a**) porous [35] (**b**) dense [36]; (**c**) asymmetric [37], and (**d**) symmetric inorganic membrane [38].

The structure and morphology of inorganic membranes have substantial effects on their performance. According to their structure and morphology, inorganic membranes are divided into porous membranes and non-porous (dense) membranes (Figure 3) [11,34].

Porous inorganic membranes may contain porous metal or ceramic supports with another additional porous layer on top with different structure and morphology. Membranes in this category have different pore shapes including straight pores with the same diameter which extend from one side to the other side of the membrane, conical shaped pores where the pore diameters at the surface are smaller than at the bottom of the membrane, pores with regular shapes and spongy structure [34]. Examples of porous inorganic membranes are glass, metal, alumina, zirconia, zeolite, carbon, ordierite, silicon carbide, silicon nitride, titania, mullite, tin oxide and mica [39,40]. 

Dense and non-porous inorganic membranes consist of either solid layers of metals (Pd, Ag, alloys) or solid electrolytes. The electrolyte layer allows diffusion of hydrogen and oxygen and also allows ions to transfer oxides through the membrane pores. Dense membranes can also have a support layer of immobilized liquid (i.e., molten salts immobilized in porous steel or ceramic supports), which fill the membrane pores creating a semipermeable layer. Some examples of dense membranes are palladium and its alloys, silver, nickel and stabilized zirconia [39,40]. Dense inorganic membranes are mainly used for the separation of hydrogen and oxygen by charged particles. The efficiency of dense membranes depends highly on the type of material, the nature of the species to be separated, and the chemical and physical interactions between the species and the membrane. The pore structure of the dense membranes depends on the synthesis protocol. 

Both porous and non-porous membranes can be symmetrical or asymmetrical. When the separation layer of the membrane cannot be distinguished in the direction of the membrane thickness the membrane is called a symmetric or isotropic membrane. The support layer in the symmetric membrane is designed to provide mechanical robustness for the membrane. On the other hand, composite or asymmetric (anisotropic) membranes are those where the top layer and the supporting layer are clearly distinguishable [41]. In these types of membranes, the majority of the flow resistance (or pressure drop) occurs mainly in the thin separation layer. The support layer is usually more porous compared to the thin layer and it does not contribute to the transport resistance of the permeate along the membrane [41]. The advantage of the asymmetric membranes is the ability to use different materials based on the nature of the species to be separated. 

Since both selectivity and permeability are important for the separation process, the properties of the separation layer is of prime importance. Generally, the material of the support layer(s) is selected based on the mechanical strength requirement and other factors such as chemical resistance and durability [14]. Preferably, the support layer must be of a more porous material in order to have a nominal gas/water transport resistance. Membranes with more than two layers in which the separating layer is overlaid on more than one support layer, the middle thin layer acts as a pressure drop regulating layer by preventing the transport of any substantial amount of particles into the pores of the underlying support layer(s). Figure 3c,d illustrates the structural differences among symmetric and asymmetric membranes.

A recent development on asymmetric membranes is the production of dual-layer hollow fiber membranes [42,43,44,45,46,47]. Hollow fiber membranes offer better advantages compared to flat sheet membranes. Some advantages are: larger membrane area per unit volume, higher flux, and better handling and flexibility for module fabrication.

## 4. Types of Inorganic Membranes

The main types of inorganic membranes include the dynamic membrane, liquid membrane, ceramic, silica, zeolite, carbon and hybrid inorganic-organic membranes. Different methods such as slip casting, sol-gel method, chemical vapor deposition (CVD) and pyrolysis have been suggested to be widely used techniques for the synthesis of inorganic membranes. There are many types of inorganic membranes currently used in water treatment and desalination. The most widely used membrane for applications in water treatment and desalination are alumina (Al_2_O_3_), titania (TiO_2_), zirconia (ZrO_2_), silica (SiO_2_) and the carbon membrane.

### 4.1. Ceramic Membrane

**Figure 4:**
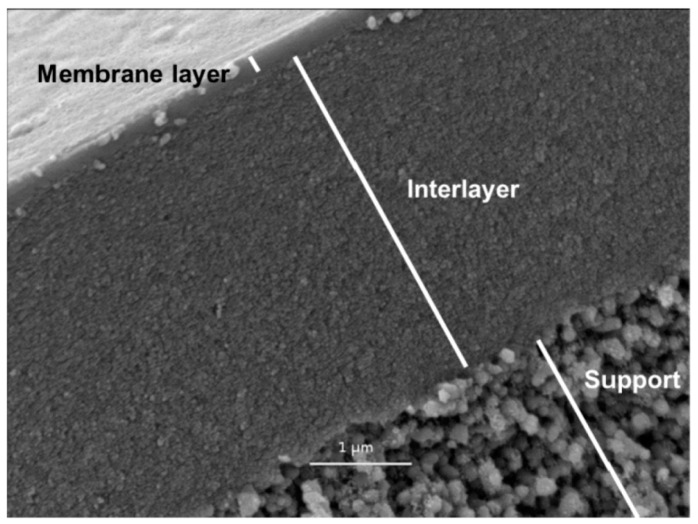
SEM micrograph of the cross-section of a ceramic asymmetric membrane structure—Reproduced by permission of The Royal Society of Chemistry [48] The membrane top layer is made of cobalt silica film with thickness of 250 nm, the interlayer is alumina with thickness of 3.5–4 mm and the bottom and substrate is coarser alumina.

Alumina (Al_2_O_3_), titania (TiO_2_), zirconia (ZrO_2_), glass (SiO_2_), silicon carbide (SiC) or a combination of these metal oxides are examples of the most commonly used materials for the fabrication of ceramic membranes. Other suitable materials include non-oxides (carbides, borides, nitrides, and silicides) and composites of combinations of oxides and non-oxides. Ceramic membranes usually have an asymmetrical structure composed of at least two, mostly three, different porosity layers; (i) a macro-porous support, a few millimeters in thickness with pore size in the range of 1–10 µm, which provides the membrane's mechanical strength while minimizing the mass transfer resistance; (ii) a meso-porous intermediate layer, with a thickness in the range of 10–100 µm and pore diameter of 50–500 nm and (iii) a top layer, with a thickness of 1 µm and pore diameter of 2–50 nm, which provides the membrane's selectivity and separation efficiency (Figure 4).

Based on their pore size, ceramic membranes are classified into three groups. The characteristics, applications, transport mechanism, advantages and disadvantages of these membranes are summarized in Table 2. 

**Figure 5:**
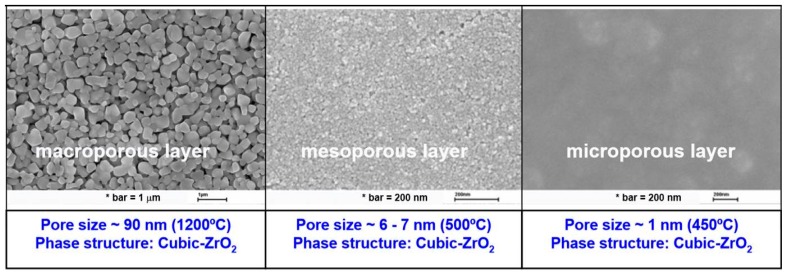
SEM image of three types of ceramic membrane with ZrO_2_ support. Reproduced with permission from [51].

Macroporous inorganic membranes generally do not provide separation functionality and are mainly used as highly permeable supports in the synthesis of composite membranes, or as a distributor of reagents, or in applications where a well-controlled reactive interface is required. An example of a macroporous inorganic membrane is the alumina membrane for applications in UF and NF. Mesoporous membranes are used in fabricating composite membranes such as γ-alumina supported on successively larger-pore layers of a α-alumina support. Mesoporous membranes are used in NF. Microporous membranes offer the potential for molecular sieving effects, with very high separation factors, and materials such as carbon molecular sieves, porous silica and zeolites have been studied (Figure 5). The most active areas of development for membrane materials currently are for the synthesis of supported thin films such as supported Pd films on porous alumina or on porous stainless steel, and supported zeolite films [49,50].

#### 4.1.1. Preparation of Ceramic Membranes

Commercially, three configurations of ceramic membranes are available in the market, namely, flat, tubular and multichannel monolith. Tubular membranes have gained significant interest due to their high surface-to-volume ratio compared to other membrane configurations. The fabrication of tubular ceramic membranes involves three main stages: (1) preparation of paste or suspension from the ceramic powder; (2) shaping the powder paste or suspension into the required geometry (i.e., tubular or flat); and (3) heat treating via calcination and sintering [52]. Other membrane modifications such as pore size control and additional layer deposition can be subsequently achieved by further heat treatment steps.

During the first fabrication step, ceramic membranes are prepared from a dispersion of fine particles named slip. Those additives that are introduced to the ceramic material at this stage affect the membrane’s microstructure and quality. Materials such as deflocculants or dispersants, in the form of various fatty acids and esters, are also added to the ceramic slip to stabilize the ceramic particles in suspension for ease of working with higher particle loading [52,53]. Some research groups reported the use of magnesium oxide to lower the sintering temperature that is required to form the alumina hollow-fiber membranes using the combined phase-inversion and sintering method [54]. Burggraaf and Cot also used a polymer binder and plasticizers to maintain the shape of the ceramic membrane precursor and achieve easy handling [55].

During the second step, the slip is deposited on a porous membrane support by a slip-casting method, at which a porous mold is used to shape the ceramic membrane into the required shape [56,57]. Due to the capillary forces, solvents are extracted from the pores during slip casting on the support layer leaving the ceramic particles on the surface. Slip casting is usually used to form hollow fiber ceramic membranes. 

Similar to slip casting, pressing is also reported for the production of flat sheet ceramic membranes [58,59,60]. In this method, a force equivalent to 10 to 100 MPa is applied on the membrane surface to produce a symmetric membrane with disc shapes. The main drawback of this method is the difficulty to fine-tune the microstructures and control the membrane thickness to diameter ratio. Moreover, the process is considered as a batch process and it is mainly used for laboratory scale fabrication of membranes [61]. 

Tape casting method is also another technique to fabricate flat-sheet ceramic membranes [62,63,64]. In this method, a suspension is first prepared and a liquid-dispersing medium and organic additive are added to the suspension to generate a ceramic paste with good suspension and pseudo-plastic behavior. The paste is then sent to a reservoir and controlled by a blade with an adjustable height to cast the membrane followed by drying in a controlled environment for subsequent processing.

Extrusion is another method to produce tubular and hollow fiber ceramic membranes [65,66]. In the extrusion process, an inorganic semi dried paste is forced through a circular orifice to take a tubular shape then compacted by applying force. 

During the third and last stage, after the ceramic membrane has taken its desired shape, the ceramic membrane precursors are fabricated. At this stage, ceramic membranes are dried and heat treated in three main stages [52], namely, pre-sintering, thermolysis and sintering. Pre-sintering takes place around 200 °C to remove water from the membrane precursors. Thermolysis is the stage where all organic components in the membrane precursor are removed. The final sintering stage is required to apply major changes to the porosity and pore size of the membrane creating the final membrane shape with the final mechanical strength [55,67].

The combined phase inversion and sintering technique is an alternative to the conventional method [54,59,68,69,70,71,72,73]. In this method, a mixed suspension of ceramic particles and polymer binder in a solvent is prepared then the phase inversion of the polymer binder takes place by solvent-on-solvent exchange, the ceramic particles are immobilized in their desired geometry by casting (flat membrane) or spinning (hollow-fiber membrane). Next, the membrane undergoes heat treatment to remove the polymer binder and organic solvents and the final membrane structure is produced. This technique is considered to be very flexible and capable of producing both symmetric and asymmetric ceramic membranes. The application of combined phase-inversion and sintering techniques to produce ceramic membranes with different ranges of porosity and pore sizes and different geometry has received enormous research attention [74,75,76,77]. 

Due to their good chemical and thermal stability, ceramic membranes, in comparison to polymeric membranes, can be easily backwashed and cleaned with various cleaning agents and sterilized at high temperatures, with no effect on their performance and/or lifetime. Krstic et al. [78] used commercial-scale MF/UF pilot plants with ceramic membranes to purify and concentrate enzymes. Other researchers also used ceramic membranes in applications such as fermentation in biotechnology industries [79,80], clarification of fruit or sugar cane juices [80,81,82] and the treatment of highly oily wastewater and degreasing baths [83,84]. 

Because of their high fabrication cost, ceramic membranes were rarely used in the production of drinking water and the treatment of municipal wastewaters [85,86,87,88], however, currently ceramic membranes are attracting more attention due to the decrease in operating and synthesis price coupled with improvement in membrane performance [89,90,91].

#### 4.1.2. Applications of Ceramic Membranes

One of the niche areas for ceramic membranes in water treatment is the use of photocatalytic materials such as TiO_2_ and composites containing TiO_2_ [92]. The presence of non-toxic, readily available, and inexpensive TiO_2_ not only provides separation but also photocatalytic ability for the decomposition of organic species/microorganisms/pollutants as well as photolysis and super-hydrophilicity to minimize fouling by organic/biological species on the surface of the membrane [93]. 

The mechanism of TiO_2_ photocatalysis is based on photo-induced charge separation on the surface of the oxide [94]. In this process, a photon with energy greater than or equal to the band-gap energy of TiO_2_ photo-excites, an electron to the conduction band (CB) of TiO_2_ causing the formation of an electron-hole pair due to the unfilled valence band (VB). Therefore, the formation of this electron hole causes degradation of organic compounds and microorganisms due to the presence of photo-induced charge carriers on the surface of TiO_2_ upon hν irradiation [14].

**Figure 6:**
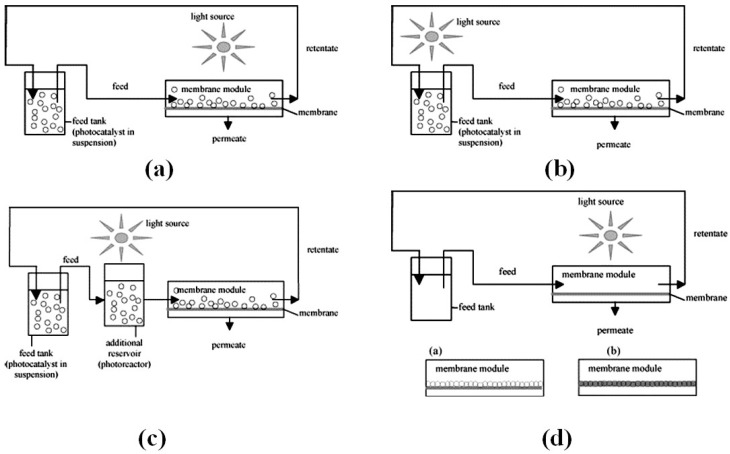
PMR utilizing photocatalyst in suspension with irradiation of the membrane (**a**); irradiation of the feed tank (**b**); irradiation of the additional reservoir (photoreactor) located between the feed tank and membrane module (**c**); and PMR utilizing photocatalyst immobilized on a membrane or within a membrane (**d**) [97]. Reproduced with permission from [97].

Photocatalytic reactions along with process utilizing ceramic membranes were studied by Alem et al. [95], Chin et al. [96], and Mozia et al. [97] for wastewater treatment. In such hybrid systems the membrane fouling is a challenge, especially in the case of MF and UF membranes and the quality of permeate is not very high, due to its passage through the membranes, even in the case of NF. Also, in many cases deterioration of the permeate flux has been observed for pressure driven membrane techniques. To solve these problem photocatalytic membranes have been used in which oxidation by hydroxyl radicals occurs on the external surface and within the pores of the membrane, whilst reactants are permeating in a one-pass flow [98]. This process is divided into two main parts: (I) reactors with catalyst suspended in feed solution and (II) reactors with catalyst support in/on the membrane [99]. Four different configurations are reported in the literature for these two groups. In the case when the catalyst is immobilizedin or on the membrane the light source is positioned above the membrane, whereas in the case of a suspended catalyst, three main configurations can be distinguished: (a) irradiation of the feed tank; (b) irradiation of the membrane module or (c) irradiation of an additional reservoir (photoreactor) which is located between the feed tank and the membrane module. For other case where the light source is positioned above the membrane module and the feed tank, the immersed UV lamps are used (Figure 6). 

Photocatalytic membranes for the photocatalytic membrane reactors PMRs have been prepared using various inorganic materials. Examples of such membranes are TiO_2_/Al_2_O_3_ composite membrane [100], asymmetric pure titania ceramic membrane [101], and TiO_2_ supported on a polymer or metallic membrane [102]. Kim et al. [103] studied the fouling behavior of ceramic membranes modified by TiO_2_ and they found that, to a large extent fouling was due to the lower adsorption amount of NOM on the TiO_2_ particles after ozonation. 

Other applications of TiO_2_ ceramic membranes are water disinfection (Removal of E. coli bacteria) [104] and the removal of organic pollutants such as methyl orange, methylene blue, Rhodamine B, humic acid, phenol, aniline and benzylamine in wastewater [105,106,107].

The treatment of oily water and produced water (PW) (saline water associated with production and exploration of oil and gas) using ceramic membranes has also been reported in the literature [108,109,110,111]. The results of these studies showed promising results when MF/UF ceramic membranes were used, reaching permeate concentrations of total hydrocarbons below 6 ppm, however the decline in permeate flux due to fouling by waxes and asphaltenes were reported by Abadi et al. [112]. Ebrahimi et al. [110] tested ceramic NF membranes to treat produced water (saline water associated with production and exploration of oil and gas) with high TOC (292 mg/L) and 2.6 mg/L of oil as a post-treatment step using a combined system of membranes. The membranes used in their studies comprised asymmetric multilayers of Al_2_O_3_ and TiO_2_ ceramic MF, UF and NF membranes in different stainless steel housings [110]. The hybrid system fully removed oil and reduced TOC by 50%. Table 3 summarizes the different ceramic membranes used for PW treatment in the literature. 

Ceramic membranes due to their robustness and chemical stability have secured their position in dealing with wastewater systems where the environment is aggressive. Wastewater treatment process using membrane bioreactor (MBR) systems having ceramic membranes have been reported [77,117,118]. Xiang et al. [118] studied the use of ceramic UF MBRs for urban wastewater treatment and the system achieved 97% reduction in the Chemical oxygen demand (COD). Sun et al. [119] used a lab-scale ceramic MBR to treat simulated high-strength wastewater with COD removal reaching 92% during the course of a prolonged sludge retention time (142 days). Tewari et al. [120] investigated and compared treating municipal wastewater using commercial nylon mesh of 30 µm pore size and 2–6 µm macroporous ceramic filters prepared from bagasse fly ash. They concluded that the ceramic filter has a higher critical flux (10–15 LMH) compared to commercial polymeric microporous membranes (1–3 LMH); and also it does not require the formation of a secondary filtration layer to ensure consistently high suspended solids retention [120].

One of the main drawbacks of ceramic membrane applications in MBR is the fouling which is affected by the membrane’s microstructure, pore sizes, and the surface roughness in immersed ceramic bioreactor systems [121]. As a general rule, the ceramic membrane with the roughest surface and largest pore size (0.3 mm) has the highest potential for being fouled compared to a smoother and smaller pore size membrane (0.08 mm). It has been investigated that using air spring reduces fouling and improves the flux in MBRs when ceramic membranes are used [122]. 

Only a few studies on membrane distillation (MD) desalination using zirconia, alumina and titanium have been reported in the literature [123,124]. Krajewski et al. [125] reported that fluorosilanes grafted ceramic membranes in the AGMD process experienced a rejection of NaCl in their study which was found to be close to 100% with the flux being 6.67 LMH. 

**Figure 7:**
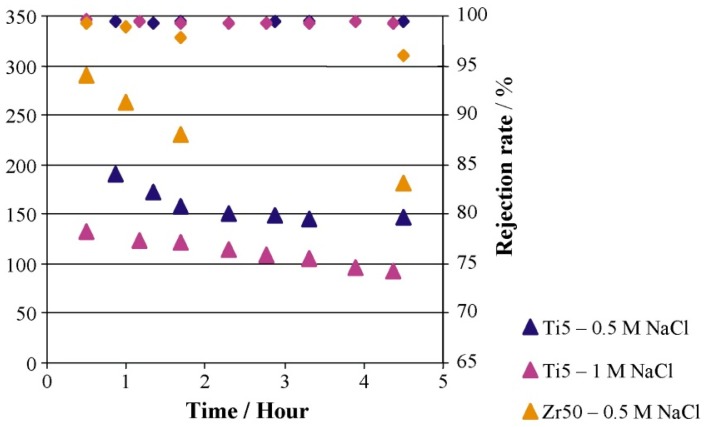
Flux (▲) on left vertical axis and rejection rate (●) on right vertical axis as a function of time in vacuum membrane distillation (VMD) of NaCl solutions (0.5 and 1 M) using Ti5 and Zr50. Feed solution: temperature of 40 °C and atmospheric pressure. Permeate side: room temperature and pressure of 3 mbar [123]. Reproduced with permission from [123].

Cerneaux et al. [123] compared zirconia and titania ceramic membranes for DCMD application to desalinate aqueous NaCl solution. The salt rejection and flux of the two membranes using two different feed solutions are summarized in Figure 7. It has been reported that the decline in the flux might be due to the driving force decrease by the concentration polarization effect caused by the increasing concentration of salt in the feed mixture during the experiment [123,126]. Also, the fact that a higher permeate flux was obtained with zirconia may be related to the existence of a lower resistance to water vapor transfer than with titania. 

Other studies have been carried out with different MD configurations and ceramic materials in the literature. A list of some of these studies is presented in Table 4. 

For the production of pure water for domestic and municipal use, the polymeric membranes market is dominant. Ceramic membranes have been used in small scale applications and a MF/UF process for the purpose of drinking water production is limited to applications such as improving the quality of tap water, recycling and reducing the volume of household wastewater, providing safe drinking water in remote areas and farms, and limited drinking water production in developing nations or during humanitarian crises [87,88].

Few studies have been reported for the utilization of MF/UF ceramic membranes to reduce the turbidity of drinking water below the standard limits. Ceramic membranes are reported to remove microorganisms, organic matter and disinfection byproduct precursors [85,88,129]. Moreover, their hydrophilic characteristics make them less affected by organic fouling compared to polymeric membranes [130]. However, high concentrations of organic matter and microorganisms in water can cause membrane fouling and hence reduce the flux. To resolve this, studies have suggested using hybrid ceramic membrane systems such as ceramic membranes for MF/UF combined with activated carbon [131] and ozonation [132] for natural organic matter (NOM) removal, the coagulation process [133] and photo-catalytic reactions [97] for disinfection. 

A new area for making an impact with ceramic membranes is investment on new membrane fabrication methods such as the combined phase inversion and sintering methods. Such hybrid methods offer an easier and simpler synthesis capable of fabricating membranes with high microstructures and varied membrane morphologies that can fit different applications. Designing the ceramic membrane such as hollow-fibre membranes is another area where it is expected to enhance the ceramic membrane market. In terms of application and usage, ceramic membranes can be used in larger-scale wastewater treatments, especially in MBRs. Two other challenges in using ceramic membranes can be summarized into reducing the capital cost of ceramic membranes by reducing the steps in the fabrication process and lowering the sintering temperature. For that, further research and development are required to decrease the sintering temperature and maintain a continuously high-quality membrane. Understanding the formation mechanisms of ceramic membrane microstructures can also assist in enhancing the reliability and reproducibility of the fabrication process, which is particularly useful when producing membranes from different ceramic materials and when the production process is scaled up. The usage and coating of different nanoparticles on ceramic membranes also improved knowledge on the integration of the particles in membrane structures for controlling membrane fouling. The use of nanoparticles in the development of low-fouling membranes opens the door for ceramic membranes to enter the nanofiltration membrane range. Although different fabrication methods using nanomaterials are available in the literature, however, a comprehensive knowledge of membrane fouling with nanoparticle-enhanced membranes in water and wastewater treatment is needed. Therefore, a knowledge of the effects of water chemistry, the nature of nanoparticles and the coating conditions on membrane performance is highly recommended. 

### 4.2. Silica Membrane

Silica membranes are widely used in industrial applications such as high temperature hydrogen separation and simultaneous reactions and separation processes. The application of silica membranes in membrane reactors has also been reported to be very successful with high yields [134,135,136,137]. 

#### 4.2.1. Preparation of Silica Membrane

Silica membranes are well known for their high selectivity, temperature resistivity, and chemical resistance. Moreover, they are less costly compared to other inorganic or polymeric membranes [138]. Amorphous silica is usually synthesized with pore size ranges from 3 to 5 Å and is suitable for water desalination applications. Several techniques have been reported for the synthesis of silica derived membranes, including sol-gel [139,140,141] and chemical vapor deposition (CVD) [136,142,143]. Extensive studies on gas separation using silica membranes prepared by both CVD and sol-gel method have been investigated, but for water applications only. Silica membranes developed via the sol-gel process have been studied [139,144,145], and the sol-gel method is mostly favored due to its simplicity and cost effectiveness, which provides more flexibility for tailoring the required porosity. In addition, the sol-gel method is frequently implemented in membrane synthesis or membrane pore modification due to its controllability and homogeneity [139,144,145]. 

**Figure 8:**
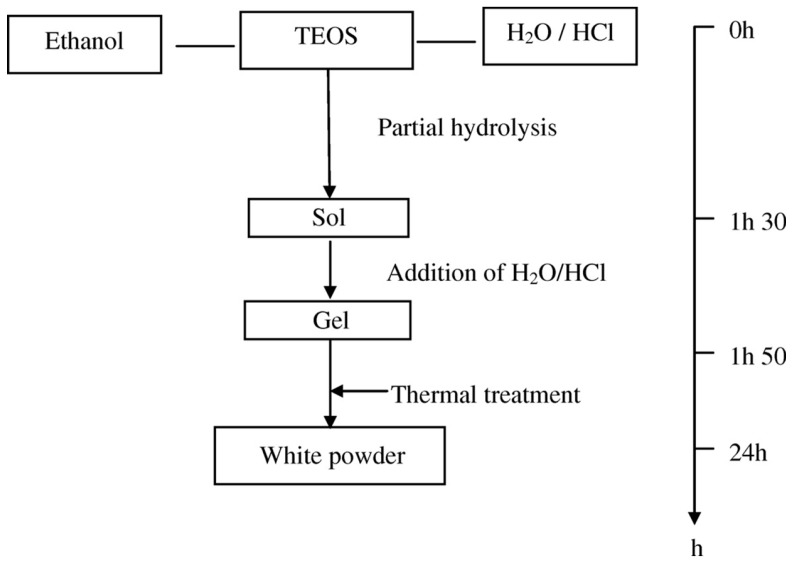
Preparation of SiO_2_ powder by the sol-gel method [154]. Reproduced with permission from [154].

The sol-gel process consists of several steps including sol preparation, gel formation, drying and thermal treatment as shown in Figure 8. Tetraethoxysilane (TEOS) is the most reported precursor for silica synthesis using the sol-gel method [146,147,148,149]. In the sol-gel method, a porous support is coated with a suitable colloidal solution (sol) followed by drying and thermal treatment [150,151,152,153]. The used sol can consist either of dense colloidal particles (for mesoporous membranes) or of polymeric-type material (for microporous membranes). Supported carbon membranes are also prepared using this method by heating precursors such as poly-furfuryl alcohol (PFA) or phenolic resins at high temperature in an inert environment leading to the formation of cross-linked nongraphitizing carbon [3]. 

The sol-gel synthesis of silica membranes consists of hydrolysis and condensation reactions with metal alkoxides to form a network. During the hydrolysis reaction, the alkoxide groups are replaced with hydroxyl groups (OH). The formed silanol groups (Si–OH) are subsequently involved in the condensation reaction producing siloxane bonds (Si–O–Si), alcohols (R–OH) and water. The desired microporous structure of the silica layer is determined not only by the reactivity and the size of the precursors but also by the appropriate selection of the precursor, water, alcohol and catalyst concentrations. Hydrolysis and condensation reactions are commonly catalyzed by mineral base or acid as follows [155,156,157]:

≡Si–OR + H_2_O ↔ ≡Si–OH + ROH (Hydrolysis) ≡Si–OR + HO-Si≡ ↔ ≡Si–O-Si≡ + ROH (Alcohol condensation) ≡Si–OH + HO-Si≡ ↔ ≡Si–O-Si≡ + H_2_O (Water condensation)

In CVD, silica deposition is formed by a gaseous reaction between a silica precursor and another reactive agent such as oxygen, water, or ozone. The reactions of the silica precursor may involve the oxidation of SiH_4_ or tetraethylorthosilicate, hydrolysis of SiCl_4_, and thermocracking [134,158]. 

The silica layer is usually placed on the porous ceramic membrane surface as a thin layer using the sol-gel or CVD technique. Examples of some widely reported ceramic layer supports are Vycor glass [159] and alumina [156,158,160,161]. 

The coating of the substrate (or support) during the sol-gel process is done by dip coating, spin coating and the pendulum methods [155]. Due to its flexibility to coat small or large substrates with various geometries, dip coating has received more attention as a process to prepare silica based membranes. Scriven [162] extensively reviewed the dip coating process and summarized the process stages into five main steps, i.e., immersion, start-up, deposition, drainage and evaporation. When the substrate is immersed in silica sol, the sol starts attaching to the substrate surface. Then, during the withdrawal step, the sol deposits on the substrate’s surface leading to the drainage of excess liquid and evaporation of the sol to form a gel on the support surface. The withdrawal speed of the substrate from the sol and the viscosity of the sol, are the two major parameters that determine the silica thin film formation. In general, the optimum withdrawal speeds are between 1 and 20 cm·min^−1^ and a dilution of 20 times the original sol volume has been reported [155]. 

Vycor glass received more interest due to its high selectivity for hydrogen separation and it is used less for water applications. The thermal expansion coefficient of Vycor glass matches that of SiO_2_ and therefore it is expected to be less affected by thermal cracking [163]. The only drawback of vycor glass is its low permeability which leads to the use of other supports such as alumina based ones, especially for water applications. 

The first silica membrane on vycor glass prepared using CVD was reported by Okubo and Inoue [164] by the oxidation of tetraethoxysilane in the pores of a tubular glass at 473 K. By changing the glass pore diameters, silica was deposited in the glass pores resulting in an asymmetrical membrane structure. For applications in gas separation, the separation factor for a helium–oxygen mixture increased by 2 fold when silica deposited on the glass surface was used. A few more publications were recently published on the same subject [165,166,167]. The only reported drawback is the small pore size (2–4 nm) of the Vycor glass support which imposes limitations on the permeability of ions and gas molecules. Due to this issue, alumina supports, which provide more permeance, were used. Alumina particles are economically cheaper compared to vycor glass and mechanically more durable [168]. The first silica membrane with alumina support was fabricated using thermal cracking of TEOS and it showed high permeability and high selectivity as reported by Morooka et al. [169,170]. 

#### 4.2.2. Applications of Silica Membrane

The utilization of silica membranes is very limited in water treatment and water separation; silica membranes with a pore size in the range of 3–5 Å are highly suitable for desalination. Microporous silica membranes usually have molecular-sieving structures with pore diameters in the range of 3–5 Å, which can be used for applications such as PV where it can act as a selective layer between the water molecules (ionic radii = dk = 2.6 Å) and the hydrated salt ions (e.g., Na^+^: dk = 7.2 Å and Cl^−^: dk = 6.6 Å) [155]. Similar to the gas separation applications, the sol-gel [139,140,171] method is more frequently used for water treatment compared to CVD [143,172]. Silicon alkoxide precursors have been utilized, but the use of TEOS is more frequently reported [146]. 

**Figure 9:**
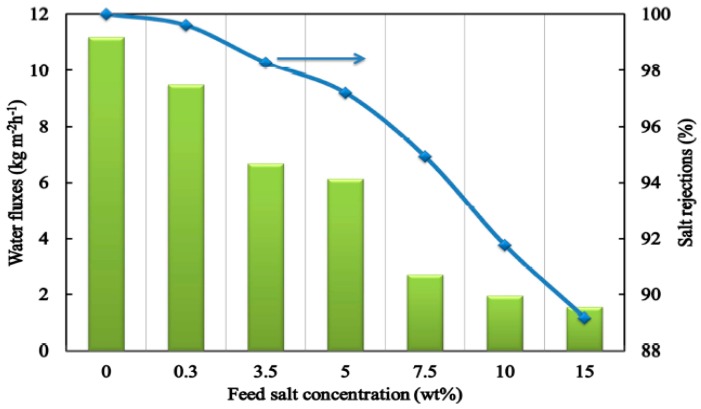
Water fluxes and salt rejections of the silica-based membrane performance as a function of salt concentration at 22 °C [175]. Reproduced with permission from [175].

Silica membranes for water treatment and desalination were first introduced in 2007 for the pervaporation of NaCl solution [173]. Later, Yang et al. [174] studied the recovery of ammonia from industrial wastewater using a molecular sieve silica membrane in pervaporation (PV). The flux of the silica membrane reached up to 3 kg·m^−2^·h^−1^. Elma et al. [175] used the silica-based ceramic membrane to measure the water flux and salt rejection at different salt concentrations. Their results showed that the water flux and salt rejection trended similarly with increasing salt concentration delivering a maximum water flux of 9.5 kg·m^−2^·h^−1^ with a rejection rate up to 99.6% and 1.55 kg·m^−2^·h^−1^ and a rejection of 89.2% for the 0.3 and 15 wt % NaCl solutions as shown in Figure 9. 

**Figure 10:**
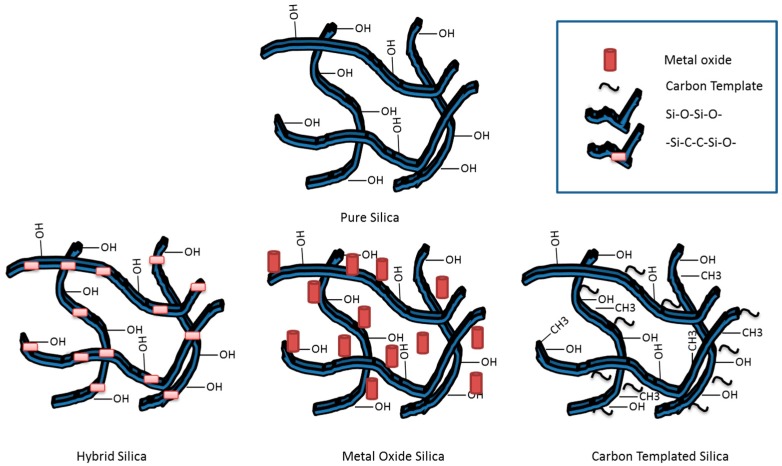
Schematic representation of different approaches for silica modification.

Due to the high affinity of amorphous silica to adsorb water molecules, one of the disadvantages of silica membranes is their structural degradation when exposed to water and hence losing their selectivity [176]. This occurs when the silica surface is rehydrated by the physisorption process with water molecules on the silanol functional group (Si–OH). The process is then followed by chemisorption with the siloxane functional group (Si–O–Si) [155]. Water, therefore facilitates the siloxane group to break and allow dissociative chemisorption by the hydrolysis reaction where siloxanes act as strong acid-base sites with a high uptake of water molecules [155,177]. This hydro-instability problem in silica membranes has generated large research activities aimed at modifying the surface properties of the silica and reducing the water-silica surface interactions. Introducing an organic template, like a methyl functional group, and a metal oxide to the silica surface of silica reduces the silica membrane hydro-instability to a great extent. Figure 10 shows the different approaches used in literature to modify the silica based membrane with different groups. 

Duke et al. [177] and other researchers [178,179] proposed a strategy to introduce non-covalently bonded, organic templates into the pure silica matrix to reduce the mobility of the soluble silica groups. The groups can be attacked by the hydrolysis process resulting in structure collapse. By introducing the hexyltriethyl ammonium bromide ionic surfactant, during the silica sol-gel preparation, a silica membrane with a non-covalent carbonized-template has been synthesized [177] and it showed hydrophilic properties with better hydro-stability [173,180].

Similarly, Wijaya et al. [181] used tetraethylorthosilicate to prepare a silica membrane as well as dodecyltrimethyl ammonium bromide and hexadecyltrimethyl ammonium bromide as surfactant for modification of the membrane to be utilized in desalination applications using low pressure pervaporation. Non-ligand carbon chain surfactants (C6, C12, and C16) as well as dodecyltrimethyl ammonium bromide and hexadecyltrimethyl ammonium bromide were used to investigate the flux and NaCl rejection for slightly, moderately and highly saline waters. The longest 16 carbon chain (C16) surfactant derived membrane showed the highest NaCl rejection up to 97% with a flux in the order of 3 kg·m^−2^·h^−1^ at 1 bar pressure difference across the membrane. The pore size of the prepared membranes remained unchanged as different surfactants were used. They concluded that the surfactant with the longest carbon chain produces membranes having the highest salt rejection, largest pore volume and surface area. The pore size of the prepared membranes remained unchanged with the different surfactants used. 

However, Ladewig et al. [182] investigated polyethylene glycol-polypropylene glycol-polyethylene glycol, tri-block copolymers as templates for hybrid carbon/silica membranes to be used in the non-osmotic desalination of seawater. The membrane was prepeared by mixing silica with 1–20 wt % PEG-PPG-PEG The membranes were tested with feed concentrations of 3, 10 and 35 ppk of NaCl at room temperature employing a trans-membrane pressure drop of <1 atm. The resulting membrane comprised double pore volume to surface area ratio when compared to the unmodified silica membrane. The porosity of the membrane even at high loading of surfactant remained unchanged and showed a microporous structure with better performance in terms of salt rejections and water fluxes.

Another strategy to avoid treating the hydro-instability of silica membranes is by incorporating terminal methyl groups (≡Si–CH_3_) using different precursors during the sol-gel process. Vos et al. [183] for the first time prepared methylated silica membranes using the hydrolysis of TEOS and methyltriethoxysilane in the presence of ethanol and water under acidic conditions. Later the membrane was calcined under an inert environment to preserve carbon on the silica matrix. The fabricated membrane showed better hydrolytic stability with degradation only occurring when the temperature was raised to 95 °C [184]. Recently, Xu et al. [185] used bis(triethoxysilyl)ethane in sol-gel synthesis to synthesize a membrane for applications in RO and PV desalination processes. The resulting membrane had a thickness of 200 nm and an effective pore size and was investigated using a range of neutral solutes such as methanol, ethanol, isopropanol, and glucose. The membranes showed quite a high rejection for neutral solutes of low molecular weight reaching up to 98.5% and lower when bigger solutes were used (95.6%). The salt rejection when NaCl was used reached 100%. 

Adding a metal oxide is another alternative to modify the silica surface for reducing hydro-instability [186,187,188,189]. Lin et al. [190] used cobalt oxide for the synthesis of silica-based membranes for water desalination. TEOS was applied during the sol-gel method with cobalt nitrate hexahydrate and hydrogen peroxide to prepare the membrane while controlling the pH in the range of 3 to 6 using ammonia. The membrane prepared at pH 5 showed a high salt rejection potential for 570 h of testing at feed temperatures up to 75 °C and NaCl salt concentrations up to 15 wt %.

The performances of different modified silica membranes for water desalination applications are summarized in Table 5, where most of the feeds tested were 0.3 to 3.5 wt % NaCl solutions to simulate brackish and sea water concentrations. 

The results summarized in Table 5 showed that with increasing temperature both the flux and salt rejection increases and on the other hand increasing the salt content caused a decline in the water flux. Thermodynamically, when the temperature of the system is increased, the vapor pressure also increased and this lead to an increase in the driving force for water permeation across the membrane. Oppositely, when the salt content increased, the vapor pressure decreased as well and this caused a decline in the driving force as well as the water flux through the membrane [191]. The purity of the water in the permeate stream is a major parameter for drinking water applications. Most of the membranes listed in the Table 5 show quite good TDS levels (TDS < 600 ppm) for slightly saline water conditions (0.3 wt %).

Based on the literature survey silica-based membranes which are used for water application are used in PV desalination and some for usage in RO processes to meet the drinking water quality requirements but these membranes need much more improvement. The RO process with polymeric membranes is a well-developed technology with years of development and research which has made the RO process dominate the large desalination industries. On the other hand RO alone cannot process all feed concentrations, in particular the pressure requirements for brine processing that might damage the membrane. Here, silica based membranes, operating under PV conditions, could be a perfect match to be used for the processing of brines or even the processing or drying of mineral salt brines. The major disadvantages of the PV process are the usage of heat for increasing the temperature of the feed to produce vapor and to increase the flux and water production. Usage of heat together with the energy requirement for water condensation makes the PV process an energy intensive process compared to the RO process which only uses pumping to pressurize the saline water feed. Using another source of heat such as waste heat from industries or solar would reduce the cost. Another benefit in the use of silica membranes is long term operational stability. Also to the present time no fouling related work has been reported for silica based membranes in the literature and more consideration and information on this issue is required. Also more research on improving the hydro-stability of the silica as well as the integrity of the membrane layer itself should continue to receive high priority. 

### 4.3. Zeolite Membrane

**Figure 11:**
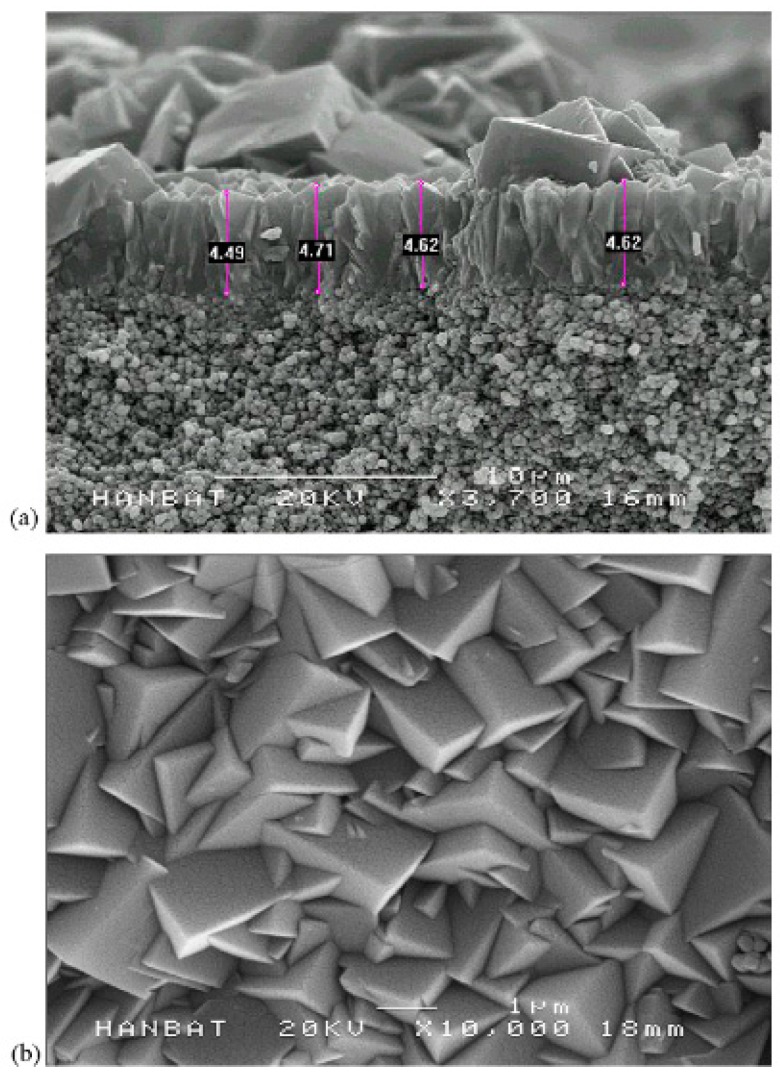
SEM images of the NaA zeolite membrane: (**a**) a cross-sectional view; (**b**) a top view [211] SEM image shows NaA and NaY zeolite crystals which are grown randomly with size of 1–2 µm. the zeolite support layer thickness is about 5 µm. Reproduced with permission from .

Both natural and synthetic zeolites are crystalline, hydrated aluminosilicates having cations in groups I and II, such as Na, K, Ca, Mg, Sr and Ba. The zeolite pores consist of rings in the framework and are selected by the number of oxygen atoms creating the ring with different structures and matrices [193,194]. The ratio between Si and Al in the zeolite structure plays a major role where most of the properties such as membrane wettability and membrane surface charge are controlled. The Al content of the zeolite structure controls the membrane’s surface hydrophilicity and water affinity [195]. Zeolite membrane thicknesses ranging from 0.5 µm to approximately 500 µm have been reported in the literature [196]. Zeolite crystals can randomly grow to the size of 1–2 µm with a zeolite layer thickness of about 5 µm (Figure 11). Different zeolite structures have been reported in the literature and examples are SOD, LTA [193,194], MOR [197,198], MFI [199,200,201], LTA [202,203], FAU [204,205], CHA [206,207], MEL [208,209], AFI [208], FER, and BEA [210]. 

Due to their unique structure and morphology along with their excellent thermal and mechanical stabilities, zeolite membranes have been utilized in many applications in membrane reactors [212], gas separation [213], fuel cells [214], pervaporation (PV) [196], and desalination [215,216]. 

#### 4.3.1. Preparation of Zeolite Membrane

Several preparation methods have been reported for the synthesis of zeolite membranes [36,217,218,219,220]. The majority of the zeolite membranes are prepared on another support to increase structural stability. The most frequently used supports in literature are generally alumina (pore diameters between 5 and 200 nm) and stainless steel tubes or discs (pore diameters between 0.5 and 4.0 nm) [196]. Studies on using a titania (TiO_2_) support with a mean pore diameter of 0.12 nm have also been reported [221]. During the formation of supported zeolite membranes, nucleation on the support followed by crystal growth to form a continuous zeolite film covering the support takes place [222]. Zeolite membranes deposited on porous inorganic supports offer a few advantages compared to the polymeric membranes such as uniformity, molecular-sized pores, high chemical and temperature stability [223,224]. 

**Figure 12:**
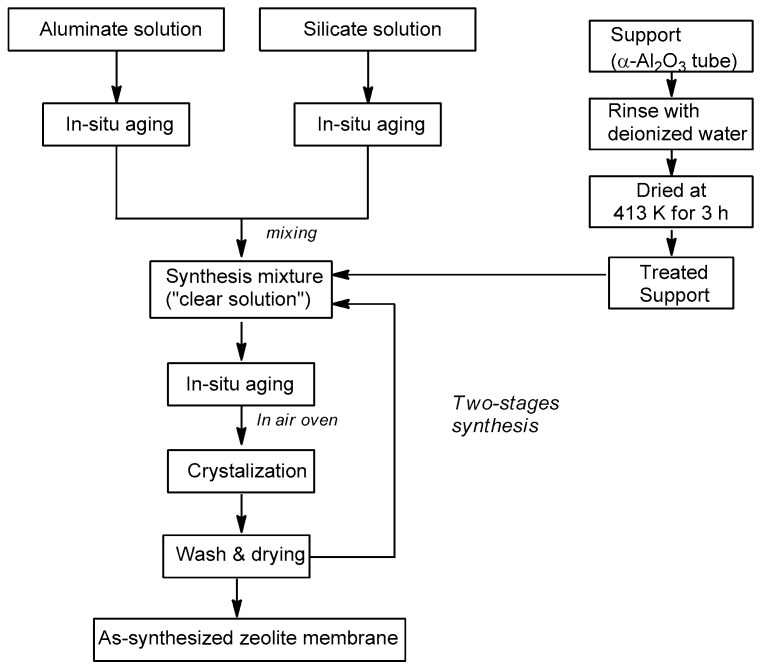
Flow diagram of the in-situ crystallization method for the synthesis of zeolite membranes [7]. Reproduced with permission from [7].

Hydrothermal crystallization is a widely used method to synthesize zeolite membranes [225,226] (Figure 12). A few studies reported the synthesis of zeolite membranes by dry gel methods [227,228,229]. In hydrothermal crystallization a gel, consisting of water, amorphous silica, a source for tetrahedral framework atoms other than Si, a structure directing organic template, and sometimes a mineralizing agent, such as NaOH, is crystallized on a porous support using an autoclave at a specified temperature and time [200,230,231]. The zeolite, when formed, possesses a crystalline structure with well-defined pores in the range of several nanometers. When the ratio of aluminum to silicon is increased, the crystal and especially the inner lumen of the pore, becomes hydrophilic with better water sorption capability inside the pores [232]. 

Sometimes seed crystals are added (referred to as two-step crystallization) to the support during crystallization to increase the number of sites available for growing zeolite crystals and also to improve the control of crystal growth [201,233]. More details on the synthesis process have been reported [196,212,234,235,236]. 

Although the hydrothermal synthesis method is simple and easy to operate, the characteristics of the fabricated membrane significantly depend on the properties of the support surface. With the hydrothermal process, the fabrication of a dense zeolite membrane is quite challenging [36,218]. The hydrothermal synthesis process requires a long crystallization time from a few hours up to a few days. As the crystallization time extends, the chance of forming impure zeolites increases too [237]. For example, a long crystallization process in the synthesis of NaA zeolite membrane, results in the production of by-products such as gmelinite, chabazite and faujasite [238]. Furthermore, due to the low heating rate and the non-uniform heating, zeolite crystals formed via hydrothermal synthesis are not uniform in size as the zeolite nuclei do not form on the support surface simultaneously [237].

Another approach in the synthesized zeolite membrane is through coating the zeolite seeds on the support surface before hydrothermal synthesis (secondary growth method). This approach is to develop a more effective method to synthesize a better quality zeolite membrane. The advantages of this method over the hydrothermal method are better control over membrane microstructure (thickness, orientation) and higher reproducibility [36,239]. With the secondary growth method, a loosely packed layer of zeolite seeds are attached to the support surface before the hydrothermal treatment. When the zeolite seeds and support are exposed to the hydrothermal process, a dense membrane is formed from the regrowth of the zeolite seeds. Common methods have been introduced to deposit zeolite seeds on the support surface such as: vacuum seeding [240,241], slip-coating [242], rub-seeding [243], and dip-coating [244,245]. Although with these methods good control of the nucleation site location and density can be achieved, this synthesis method is complicated and involves multi-steps and in some cases the use of a binder, which might affect the layer properties [246,247].

Recently a newer approach has been introduced through the continuous flow synthesis method to synthesize inner-side zeolite membranes [217,248,249]. It has been reported that due to the low accessibility to the lumen of tubular supports, growing a layer of membrane on the inner side of the support is a stimulating task for the synthesis of zeolite membranes [250]. In the continuous flow synthesis process, the reactants are supplied to the support surface continuously. The continuous synthesis process is more energy efficient due to less energy consumption needed for repeated heat-up and cool down in batch crystallizers. In terms of capital cost, the continuous process needs fewer requirements for equipment for the same production rate. Finally, this method is capable of producing a more uniform product because of the readily controlled operating conditions [251]. 

Microwave synthesis is another approach for the synthesis of zeolite membranes [252,253,254,255]. Compared to the conventional hydrothermal synthesis, microwave synthesis has a shorter synthesis time, broader synthesis composition, small zeolite particle size, a narrow particle size range distribution and high purity [256]. Due to the direct supply of an electromagnetic field to the material, thermal energy is transferred more efficiently through convection, conduction and radiation [257]. However, the number of publications using microwave synthesis is very limited. Li et al. [258] investigated the synthesis of the LTA zeolite membrane by using the “in-situ aging—microwave synthesis” method. The membrane was tested for pervaporation and failed at high water concentrations but showed excellent long-term stability in vapor permeation. 

#### 4.3.2. Applications of Zeolite Membrane

Most zeolite based membranes have been used for gas separation applications [196,223] whereas there are very few reviews on water desalination discussed in the literature [212,259,260]. Desalination based on pervaporation using zeolite membranes appeared first in 2008 when water contaminated with radioactive material was separated using a NaA zeolite membrane [261]. 

**Figure 13:**
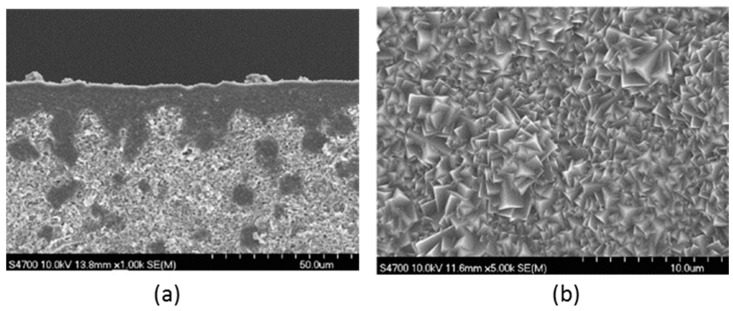
SEM images of (**a**) cross-section and (**b**) top-surface of NaA zeolite membrane [266]. The support material is porous glass-ceramic (silica) composite the intermediate layer is grown non-uniformly. Reproduced with permission from [266].

**Figure 14:**
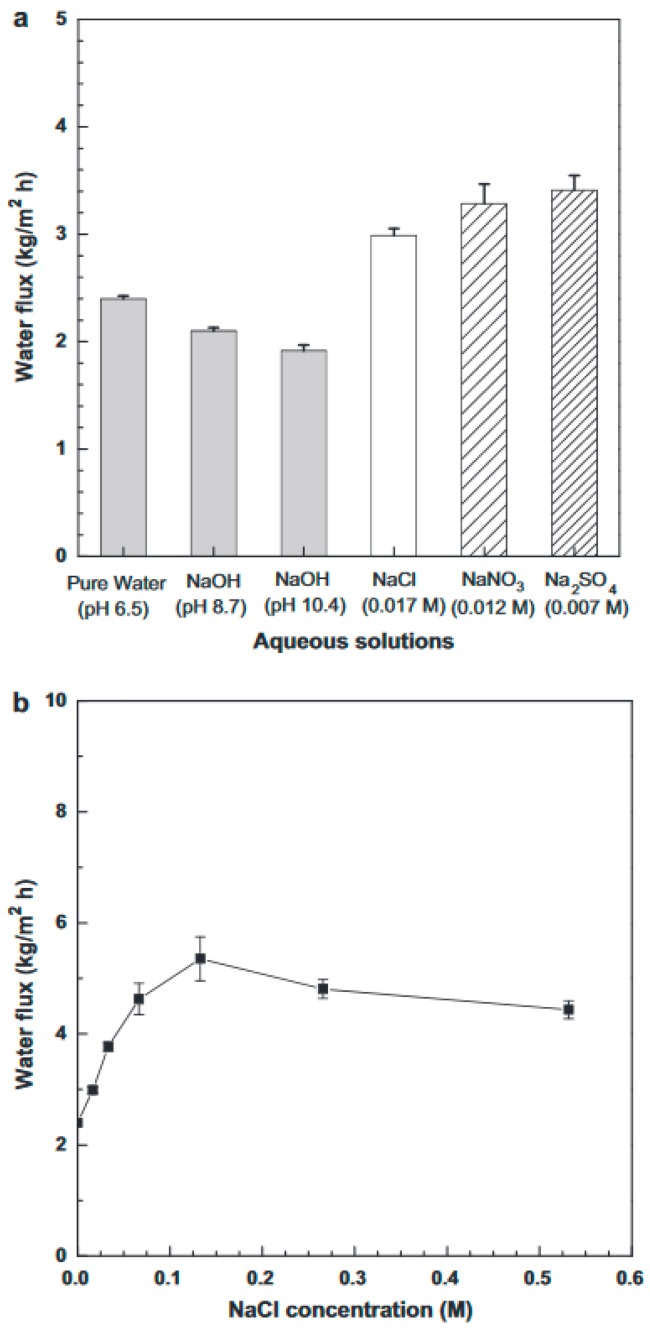
Pervaporative fluxes of NaA zeolite membrane with (**a**) pure water, NaOH (pH 8.7 and 10.4), 0.017 M NaCl, 0.012 M NaNO3 and 0.007 M Na_2_SO_4_ solutions and with (**b**) various NaCl solutions [266]. Reproduced with permission from [266].

Thereafter, extensive research has been undertaken for testing zeolite membranes for water applications. Li et al. [262] for the first time used MFI structured zeolite membranes (average pore diameter of 5.6 Å) in RO desalination of 0.1 M NaCl solution at an operating pressure of 2.4 bar and the results showed 80% salt rejection with flux reaching up to 0.11 L/m^2^h. Kazemimoghadam [263] fabricated a HS zeolite membrane for RO applications and studied the effect of operational conditions on membrane structure and performance. It was found that the permeate flux increases with increasing feed temperature and feed rate. The tested HS zeolite membrane had a permeate flux of 4 L/m^2^h. Liu et al. [264] used a MFI type zeolite membrane to desalinate 0.1 M NaCl solution with 100 ppm organic contaminant (toluene and ethanol) at 2.76 MPa. It was shown that 94% salt rejection was achieved and 99.5% of the organics were removed. The water flux was reported to be around 0.03 kg/m^2^h. Fathizadeh et al. [265] synthesized NaX nano-zeolite membrane by modifying the top PA layer of the polyamide RO membrane using the surface coating method. The modified membrane showed higher water permeability and was thermally more stable than the pure zeolite membrane. The surface chemistry of the modified membrane showed excellent enhancement in terms of roughness, contact angle, and solid-liquid interfacial free energy. However, salt rejection decreased drastically due to the increase in pore size. Cho et al. [266] used NaA zeolite membrane for pervaporative water desalination at different salts concentrations. The SEM of the membrane is shown in Figure 13. High ions rejection (more than 99.9%) was achieved with different salts (Figure 14), while boron removal reached up to 79%. 

Other studies on water desalination with different types of zeolite membranes can be found [264,267,268,269,270], including ZSM-5 [271], zeolite A [248,272], mordenite [273] and zeolite Y membranes [274,275].

In general, the performed studies indicate that for desalination applications, zeolite membranes with their excellent molecular sieving and viable adsorption and diffusion capabilities can be a good alternative for RO processes. Zeolites due to their cation exchange ability can be a competitive option for other desalination processes where removals of dissolved cations are essential [276]. Zeolite membranes are mostly very costly and therefore the industrial uses of zeolite membranes for desalination processes are limited. 

Zeolite membranes have been also tested for ethanol, butanol, and IPA dehydration and they showed promising results with flux and removal efficiency being higher than those of polymeric membranes [277,278,279,280]. Zhou et al. [281] used ZSM-5 zeolite membrane with 40 µm thickness to separate alcohol from water. Chen et al. [282] fabricated silicalite-1 membranes to separate methanol–water, ethanol–water, 2-propanol–water and 1-propanol–water mixtures. 

The dehydration of alcohols and other organic compounds by pervaporation using a large scale plant operated with zeolite membranes has been reported in the literature [226].

An economic analysis done by Kaminski et al. [283] for ethanol dehydration by different methods for systems with a daily capacity of 30 tonnes/day showed that vapor permeation, pervaporation with zeolite membrane, distillation and adsorption required a total operating cost of US$ 15.75/tonnes, US$ 12.6–16.6/tonnes, US$ 31.95–45.65/tonnes and US$ 36.3/tonnes respectively.

For industrial water treatment where the organic content is high, zeolite membranes can be a good candidate. Studies are available in literature for different membranes such as NaX for the removal of 1,3-propanediol from glycerol and glucose in water by pervaporation [284], NaY for water/ethanol (10/90 wt %) and methanol/MTBE (10/90 wt %) mixtures separation [274], and separation of p-xylene/m-xylene mixture using ZSM-5 [285,286,287]. An industrial scale zeolite based desalination plant with 16 modules, each of which consists of 125 pieces of NaA zeolite membrane tubes is available in Japan and was developed by Mitsui Engineering and Shipbuilding Co. Ltd. It is based on NaA zeolite membranes for pervaporative dehydration of organic-water mixtures [226]. 

Synthetic zeolite RO membranes were used to desalinate produced water during oil exploration with TDS reaching up to 181,000 ppm [288]. The results showed that by using a zeolite membrane at an operating pressure of 55 bar, the TDS of water reduced to 11% by weight with the flux reaching 0.018 kg/m^2^ h. Studies are available in the literature for different membranes such as NaX [284], NaY [274], and ZSM-5 [285,286,287] where applications for treating industrial water have been used. Leo et al. [289] studied the effect of fouling by humic acids on zeolite membranes using a SAPO-44 zeolite filler. They prepared a PSF/PVA/SAPO-44 UF membrane and the results showed that the water flux increased by almost double compared to the PSF polymeric membrane. A fouling study revealed that 80% of the permeate flux for the membrane remained constant during the filtration experiment.

Although studies on zeolite membranes show promising results, it is very challenging to fabricate a defect free zeolite membrane with a practically useful thickness. Therefore, extensive work on the orientation of microcrystals in the zeolite layer, enhancing economic feasibility of the zeolite membrane, scaling up opportunities, and stability tests should be done to enhance the zeolite membrane performance and increase the market share for water treatment applications [222,290]. 

### 4.4. Mixed Matrix Membranes (MMM)

In general, mixed matrix membranes (MMM) are those membranes having inorganic nanoparticulate materials (the “filler”) incorporated into a macroscopic polymeric material (the “matrix”). The main objective of fabricating MMM is to overcome some of the drawbacks of polymeric and inorganic membranes by combining high mechanical properties of the inorganic filler with the superior processability and low cost of polymeric materials [291,292]. MMM are prepared by several methods, including phase inversion and surface coating [293,294]. 

#### 4.4.1. Preparation of MMM

Recently extensive research has been carried out to incorporate inorganic nanoparticles such as SiO_2_ [295,296,297,298,299], silver (Ag) [300,301], ZrO_2_ [302,303,304], Fe_3_O_4_, Al_2_O_3_ [302], TiO_2_ [302,305,306,307,308,309,310,311], ZnO [312,313], zeolites [289,314,315], organo-selenium compounds [316], and carbon nanotubes (CNTs) [317,318] into polymeric membranes. Table 6 summarizes a few studies which have used different inorganic fillers in the polymeric structure for their application in water treatment. 

#### 4.4.2. Applications of MMM

**Figure 15:**
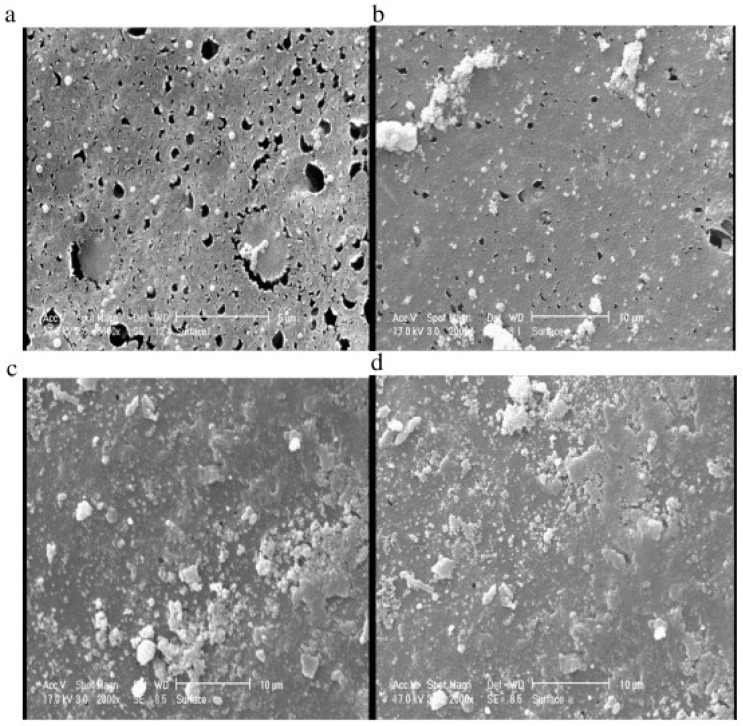
Surface SEM images of neat and TiO_2_ deposited PVDF/SPES membranes: (**a**) neat membrane; (**b**) 0.1 wt % TiO_2_; (**c**) 0.5 wt % TiO_2_; (**d**) 1 wt % TiO_2_ [7] TiO_2_ nanoparticles are distributed uniformly on the surface of membrane and pores are filled with the NPs. Reproduced with permission from [7].

TiO_2_ is one of the NPs which have attracted much interest due to its antibacterial properties when used with UV irradiation. Rahimpour et al. [334] used a PVDF/sulfonated polyethersulfone (SPES) blend UF membrane and coated with different concentrations of TiO_2_ nanoparticles on the membrane surface (Figure 15). Although the synthesized membrane showed lower flux compared to non-coated PVDF membrane, the contact angle and anti-fouling behavior improved and the flux stability of pure water was enhanced significantly. Mollahosseini and Rahimpour [335] enhanced the anti-fouling properties of PS UF membrane by coating it with a TiO_2_ layer. The resulting membrane appeared to be smoother and thicker with less fouling tendency towards Bovine serum albumin solution. 

Similarly, SiO_2_ nanoparticles were also used for membrane modification to improve the membrane hydrophilicity and the mechanical properties. Yu et al. [296] used grafting of SiO_2_ nanoparticles modified with N-halamine on PES membrane to develop a novel hydrophilic PES UF membrane. The hydrophilicity of the membrane was significantly enhanced by the addition of SiO_2_ nanoparticles creating a slight change in the membrane structure such as a decrease in the thickness of the skin layer, an increase in the connectivity of the pores between the sub-layer and the bottom layer, and the finger-like microvoids enlarged across the membrane thickness. Another study used a PVP-grafted SiO_2_ NP membrane for UF application [336]. The water flux of a membrane containing 1 wt % PVP-g-silica was reported to be 2.3 times higher than that of the neat PSF membrane. The hydrophilicity of the PSF/PVP-g-silica membrane was also reported to increase with increasing PVP-g-silica content. The PSF/PVP-g-silica membranes exhibited enhanced fouling resistance in fouling experiments using nonionic surfactants with a slight decrease in salt rejection efficiency. Moreover, the contact angle of the membrane increased with increasing SiO_2_ loading. Other studies on water desalination showed that membranes modified with ZnO nanoparticles are economically more feasible compared to those modified with SiO_2_ or TiO_2_ due to the catalytic activities of ZnO [312,337].

Graphene oxide (GO) nanosheets also received great attention recently for their unique properties, such as good hydrophilicity, ease of modification, distinctive structural characteristics, high mechanical strength and negligible thickness. Zinadini et al. [338] prepared NF PES membranes by blending GO nanoplatelets in a PES matrix. The modified membranes were tested with a dead-end permeation cell, under an operating pressure of 0.4 MPa, pH = 6 and dye concentration of 30 mg/L. the modified membrane showed the rejection capability of the prepared GO blended membranes was higher than that of the unfilled PES membrane. It has been reported that the GO nanomaterial can induce a surface negative charge throughout the entire pH range. The blended membrane also showed higher water flux, lower contact angle, and better dye removal compared with the unfilled PES membrane. Wu et al. [339] fabricated a PS membrane with SiO_2_ and GO by depositing silica nanoparticles SiO_2_ on GO nanosheets by in-situ hydrolysis of TEOS, which also showed very promising antifouling properties towards protein (egg albumin) rejection and better water flux with high rejection (98%).

Ag nanoparticles were grafted on PA membranes in different studies [270,340,341]. The PA membrane was synthesized by an interfacial polymerization on the surface of a PES support layer between the aqueous and organic phase. The organic phase was made up of 1,3,5-benzene tricarbonyl chloride and dissolved in 1,1-dichloro-1-fluoroethane together with silver nanoparticles at 10 wt % relative to the polymer. The permeation flux of the solution through the membrane and salt rejection was carried out using a 2000 ppm aqueous MgSO_4_ solution as a function of the operating pressure where the produced membrane showed an identical flux to that of the pure PA membrane but with enhanced antimicrobial action against Pseudomonas.

Examples of other inorganic filler materials which have been used in the polymeric membranes for water applications are carboxylic MWNTs into PA [342], oxidized MWNTs in PVA [343], amine functionalized MWNTs in PA [344], Cu NPs in PES [345], Se NP into PES [345], cyclodextrin into PSU [346], and polypyrrole into PSU [347]. 

Progress in the enhancement and development of MMM has been very rapid in recent years and all new developments cannot be covered in this section. Tuning and modification of the physicochemical properties of membranes and the method of incorporating nanomaterial into polymeric substrates have provided membranes with extraordinary properties to be used for water treatment and desalination applications. There are still challenges in this area to optimize the design of the MMM for industrial and practical applications. Examples of such challenges are: the development of fundamental understanding on how nanomaterials effect membrane structures and then correlating this information with the membrane performance, studies on better NP dispersion into polymeric matrices, solving the aggregation and agglomeration issues of NPs into the polymeric matrix, the compatibility of nanofillers with polymers, which will determine membrane performance, and the stability of nanofillers within the host polymer, and finally practical and real applications of nanocomposite membranes for water treatment. Although a lot of work has been reported in the literature no real industrial scale or practical applications of MMM have been reported. Therefore, more work is needed to evaluate the cost-effectiveness of large scale membrane fabrication [348]. 

### 4.5. Dynamic Membrane

Another type of inorganic membrane which was studied in 60s and 70s is called the dynamic membrane (DM) [349]. In this type of membrane, a colloidal support surface is formed on the microporous support layer when filtering a dispersion containing the suspended inorganic or polymeric colloid. This layer acts as an active separating layer, which in, time would dissolve and disappear. 

**Figure 16:**
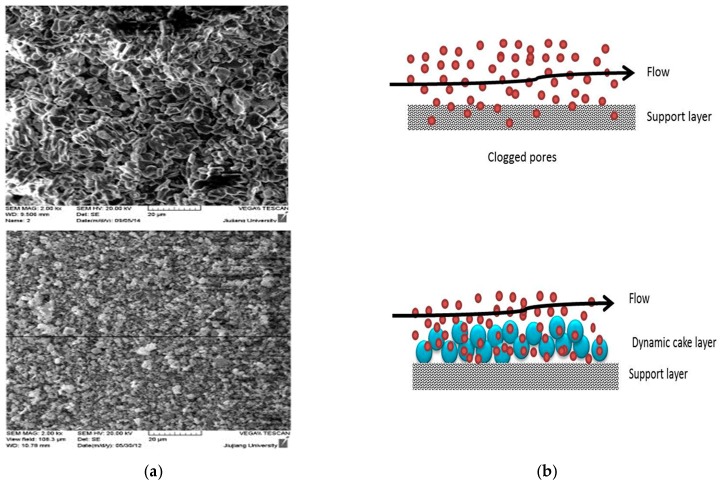
SEM images of the surface of (**a**) ceramic inorganic membrane and (**b**) Kaolin dynamic membrane [351]. Kaolin dynamic membrane show smaller pore size in the range of 0.18 μm where ceramic membrane has pore size in range of 0.83 μm. Reproduced with permission from [351].

**Figure 17:**
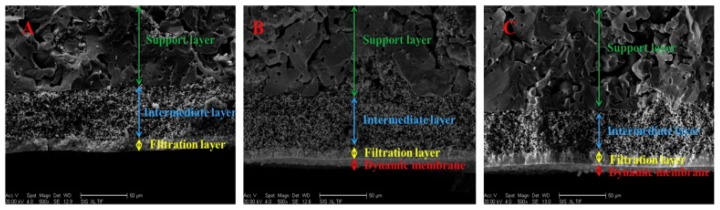
(**A**) Cross-section SEM of support ceramic membrane; (**B**) pre-coated composite membranes and (**C**) self-forming composite membranes [350]. Reproduced with permission from [350].

Dynamic membranes are categorized into two main classes, namely, self-forming DMs (SF-DM) and pre-coated DM. The SF-DM is formed by materials present in the separation media such as suspended solids in the water while a pre-coated DM is formed by passing a colloidal solution of one or more materials over the surface of a porous membrane (Figure 16) [350]. The SEM image in Figure 17 illustrates the difference between a regular ceramic membrane, SF-DM, and a pre-coated DM. 

This technique was commonly used for the support for materials such as porous stainless steel, carbon or ceramics. Materials such as mesh, non-woven fabric, porous stainless steel, carbon, ceramics, and woven filter-cloths are examples of DM support layers which can act as the supporting layer instead of microfiltration (MF) or ultrafiltration (UF) membranes [352,353]. The main purpose of the mesh, which is made of connected strands of metal, fiber or other flexible/ductile material, is to provide a permeable barrier. These meshes might sometimes be problematic especially in MBRs and wastewater treatment systems, since there might be inefficient sludge accumulation (for self-coated DM) due to their flat structure [354]. A woven cloth is made of monofilament and/or multifilament yarn which are made by extruding synthetic filaments with smooth surfaces. Whilst the multifilament fibers are made from a group of fine monofilament fibers spun together to form the individual yarns that are eventually woven together [349], the non-woven cloth is a web of natural or man-made fibers that are bonded to each other [355]. A list of possible support materials with their applications in the literature have been summarized in Table 7.

On the other hand different materials have been reported in literature for the dynamic layer. A list of different materials which have been used in the literature and their applications in water treatment are tabulated in Table 8.

Dynamic membranes have been studied for different processes in water treatment applications since 1960s. Reverse osmosis applications, such as desalination of brackish water, were found to be difficult to provide a consistent performance and the added cost of the consumables makes the process unattractive economically. Therefore, only limited applications for recovering polyvinyl alcohol in the field of textile dyeing is commercially practiced [349].

The first study on physical DMs was investigated by Marcinkowsky et al. [367] who used a zirconium oxychloride (ZrOCl_2_) membrane for salt rejection using an RO process. After that, a few more studies were conducted by researchers on salt rejection using RO membrane to study the performance of such membranes [368,369,370]. The application of DM for UF processes started in 1980s for wastewater treatment applications for the removal of dyes and proteins [371]. Some researchers have also tested dynamic UF membranes in the food industry [372,373]. Due to their high cost and low permeability such applications did not receive great attention and further developments and scale up have stopped. 

By the 1990s, the first MF application of DM was tested for wastewater treatment by a few researchers [357,361,366,374,375]. The good results and high performance proved DM can be a viable possibility for oil/water separation [358,376]. Also a few other researchers tested applications of aerobic and anaerobic wastewater treatment using DM filtration and promising results with acceptable removal efficiencies for SS, biological oxygen demand (BOD) and chemical oxygen demand (COD) were obtained [352,353,377]. Therefore, dynamic membranes seem to be a viable and economical technique for small wastewater treatment systems where investment and operational costs are minimal and ease of operation is required. 

### 4.6. Liquid Membrane

**Figure 18:**
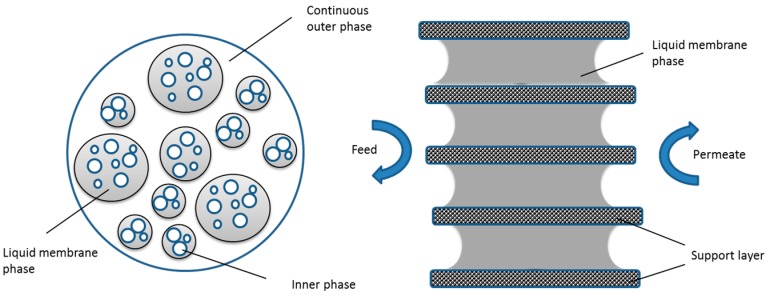
Schematic diagram of liquid membrane with an emulsion (**left**) and immobilized lamellae (**right**).

Liquid membranes (Figure 18) are another type of dense organic membranes where a liquid complexing or carrier agent reinforced or immobilized in a rigid solid porous structure act as the separating transport medium [378,379,380]. The liquid carrier agent fills the pores of the support medium and reacts with the permeate on the feed side. The complex formed diffuses across the membrane/support structure and then releases the permeate on the product side and at the same time recovers the carrier agent which diffuses back to the feed. In such membranes separation is achieved through complexing reactions and diffusion. The main applications of such membranes are in gas separation or coupled transport where metal compounds are separated through ion transport. Although liquid membranes are highly selective, they lack physical stability and this made them to be not commercially viable. 

Liquid membranes are classified into three different categories namely, bulk, emulsion and supported liquid membranes (SLM). Bulk liquid membranes (BLM) consist of a stripping phase and an aqueous feed phase, which are separated by a water-immiscible liquid membrane phase in a trap with a U shape. Supported liquid membranes (SLM) contain an organic liquid which is fixed into some small pores of a polymer support and is kept there due to capillary forces. Emulsion liquid membranes are based on water immiscible emulsion dispersed in an organic phase. The emulsion is encapsulated and emulsified into the membrane phase which is an inorganic material. The emulsification occurs due to the presence of a surfactant [381]. 

The applications of these liquid membranes and their family in the literature are limited to the extraction of some heavy and precious metals from water. Extraction of copper [382], zinc [383], nickel [384], alkali metals [385], precious metals [386], and rare earth metals [387] are a few examples of the usage of liquid membranes in water treatment. 

Although a few promising results on liquid membrane selectivity and permeability have been published in literature, the development stage of liquid membranes is far from that of conventional organic or inorganic membranes and therefore cannot compete with the well-developed membrane technologies for large scale applications. In fact, there have been only a few pilot studies reported in the literature in which liquid membranes were utilized [388]. 

## 5. Conclusions and Future Outlook

Membrane technology is a cost effective and simple process that is widely used in the separation industries for different applications especially in water processes and applications of water treatment. 

This state of the art review has focused on the development stages of inorganic membranes in the separation industries with more focus on water treatment applications. A comprehensive comparison between organic and inorganic membranes has been reviewed, analysed and critically discussed. Different types of inorganic membranes have been introduced from the synthesis perspective as well as their application in the water treatment industries. 

Inorganic membranes are widely used due to their superior properties such as mechanical and thermal stability which are lacking in polymeric membranes. So far, most of the studies are dedicated to the design and preparation of various inorganic membranes. Little attention has been given to deep water separation mechanisms and the understanding of how the water molecules pass through the membrane pores. Giving more attention to such aspects areas may lead to a better theoretical understanding for designing high-performance inorganic membranes for water treatment applications. 

Dynamic membranes and their applications in water treatment and desalination have been researched generally but comprehensive detailed research on their usage for aerobic MBRs, treating municipal sewage and as primary settlers to remove the particulate organic matter with a high efficiency in municipal wastewater treatment plants needs to be studied in more depth. Furthermore, to evaluate the long term performance, reliability, and operability of the DM applications need further research, preferentially in combination with other areas such as fluid dynamics and sludge properties for full-scale applications. 

Liquid membranes are very cheap and are the most efficient membranes as they do not operate under any external driving force such as voltage or pressure but are based on the difference in chemical energy as a driving factor of the process. More research on their application for hollow fiber mode is required as they can be used as a porous support whereby they can increase the membrane surface per unit of volume and maintain good membrane stability.

Undoubtedly, ceramic membranes can be considered as one of the most promising inorganic membranes so far and can become the new playmaker in the desalination and water treatment industry. Comprehensive research and in-depth studies on easier and cheaper synthesis and fabrication methods to reduce their price will definitely change the membrane market for water treatment applications. Overcoming challenges such as reducing the capital cost of ceramic membranes by reducing the steps in the fabrication process and lowering the sintering temperature plus understanding the formation mechanisms of ceramic membrane microstructures, the reliability and reproducibility of the fabrication process, incorporating different coatings of different nanoparticles on ceramic membranes and a good knowledge of the effects of water chemistry and its interaction with ceramic membranes can help progressing this area. 

The use of silica membranes for water applications might be limited due to the high affinity of amorphous silica to adsorb water molecules, and their structural degradation when exposed to water. However, finding novel ways to protect the structure of silica based membranes through incorporating organic templates or metallic nanoparticles can help such membranes to enter the membrane market. 

One of the main challenges, despite their good application for zeolite membranes, is to fabricate a defect free membrane with a practically useful thickness. Therefore, more work on synthesis and orientation of microcrystals in the zeolite layer, enhancing the economic feasibility of zeolite membranes, scaling up opportunities, and stability tests are suggested to enhance the zeolite membrane performance and increase the market share for water applications. 

Finally, but significantly mixed matrix membranes (MMM) offer a very unique platform for both flux and performance enhancement as well as anti-microbial properties. One of the excellent advantages of MMM is their significant performance with very minimal changes to current polymeric manufacturing processes. Membranes with a mixed matrix of organic and inorganic materials show improvement in terms of robustness, surface hydrophilicity, and anti-fouling properties. Similar to any other technology at an early stage, MMM research needs to develop some commercial applications as well as cost analysis to become a mature technology for water applications. 

## Figures and Tables

**Table 1:** Comparison between organic and inorganic membranes.

Properties	Polymeric Membranes	Inorganic Membranes
Material	Rubbery or glassy type membranes based on the operating temperature [22]	Inorganic materials i.e., glass, ceramic, silica, etc. [11]
Characteristic	Rigid in glassy form and flexible in rubbery state [23]	Chemically and thermally stable, mechanically robust, operational under harsh feed condition [11]
Advantages	Cost-effectiveness, good selectivity , easy processability [1]	Withstand harsh chemical cleaning, ability to be sterilized and autoclaved, high temperature (up to 500 °C) and wear resistance, well-defined and stable pore structure, high chemical stability, long life time [11,21]
Disadvantages	Fouling, chemically nonresistant, limited operating temperature and pressure, short life time [24,25]	Fragile, rigid [26]

**Table 2:** Types of ceramic membranes based on their pore size and permeation mechanism [49].

Porous Membrane	Pore Size Diameter, nm	Applications	Permeation Mechanism	Advantages/Disadvantages
Microporous	<2	Gas separation + NF	Molecular sieving	Low pore size diameter with a potential use in molecular sieve separation
Mesoporous	2–50	NF + UF and gas separation	Knudsen diffusion	High permeability and low selectivity. Used in the synthesis of composite membranes.
Macroporous	>50	UF/MF	Poisseuille flow	High permeability as a support in the synthesis of composite membranes, or as a distributor of reagents

**Table 3:** Different studies on produced water (PW) treatment using various ceramic membranes.

Ceramic Membrane Material	Application Type	Flux	Removal Efficiency	Reference
Al_2_O_3_	MF/UF	118–125 LMH	99% (oil and turbidity) and 100% (TSS)	[113]
ZrO	UF	600 LMH	90% (oil and turbidity) and 100% (TSS)	[114]
Al-ZrO	NF	190–250 LMH	95% (TDS)	[115]
α-Al_2_O_3_	MF	250	95% (oil)	[116]
Al_2_O_3_/TiO_2_	MF/UF	3.4 to 3300 LMH	99.5% (oil) and 49% (TOC)	[110]

**Table 4:** Summary of studies related to the use of ceramic membranes in membrane distillation (MD) applications.

Ceramic Material	Temperature Difference (°C)	Flux (kg/m^2^h)	Rejection %	Reference
TiO_2_	65	13.44	92.3	[123]
	85	57.74	99.8	
Clay Alumina	20	5.48	99.1	[127]
	60	98.66	99.96	
ZrO_2_	55	15.7	100	[124]
	85	82.7	100	
Al_2_O_3_	58	18.2	100	[124]
	90	129.5	100	
B-Sialon	50	100 LMD	99	[128]
	80	290 LMD	99	

**Table 5:** Flux and rejection of modified silica membrane in desalination tests with NaCl solutions.

Membrane Type	Operating Condition	Feed Concentration	Water Flux (kg/m^2^h)	Rejection	Ref.
**Carbonized silica membranes**	20 C, 7 bar, 5 h	0.3–3.5 wt %	2.1–1.9	99.5%	[173]
	20 C, 1 bar	0.3–3.5 wt %	3.2–1.4	89%	[181]
	20 C, 1 bar	0.3–3.5 wt %	2.8–1.6	89%	[181]
	20 C, 1 bar	0.3–3.5 wt %	3–2	94%	[181]
	20 C, 1 bar, 12 h	0.3–3.5 wt %	1.5	95%	[182]
	20 C, 1 bar, 12 h	0.3–3.5 wt %	6.3–4.9	92%	[182]
**Silica membranes with incorporated metal oxide**	20 C, 1 bar, 570 h	0.3–15 wt %	0.4–0.3	99.8%	[190]
	50 C, 1 bar, 570 h	0.3–15 wt %	0.9–0.35	99.7%	[190]
	75 C, 1 bar, 570 h	0.3–15 wt %	1.8–0.55	99.7%	[190]
**Methylated silica membrane**	30 C, 1 bar	0.2	3	99%	[192]
	90 C, 1 bar	0.2	3.4	99.9%	[192]
	20 C, 1 bar, 5 h	0.3–3.5 wt %	4.7–2.5	88.3%	[173]

**Table 6:** Summary of studies on mixed matrix membranes (MMM) with different inorganic fillers.

Filler	Polymeric Support	Application	Synthesis Method	Advantage	Reference
TiO_2_	PVDF	-	Directional melt crystallization	Improved the hydrophilicity	[319]
GO/TiO_2_	PVDF	UF	Solution casting and phase inversion method	Improved photo-catalytic activity	[320]
TiO_2_	PVC	UF	Non-solvent induced phase separation method	Improved the hydrophilicity	[321]
TiO_2_	PES	UF	Phase inversion method	Improved the hydrophilicity	[302]
SiO_2_	PES	MF	Combination of vapor induced phase separation and non-solvent induced phase separation	Improved the hydrophilicity	[322]
SiO_2_	CA	UF	Phase inversion	Improved the hydrophilicity	[323]
SiO_2_/GO	PVDF	UF	Thermally induced phase separation method	Improved the hydrophilicity	[324]
Al_2_O_3_	PPY	UF	Phase inversion	Improved adsorption capacity	[325]
Al_2_O_3_	PES	MBR	Phase inversion	Improved the hydrophilicity	[325]
Fe_3_O_4_	PVA; PES; PVC	UF/MF	Phase inversion	Magnetic property; improved Hydrophilicity	[326,327,328]
ZrO_2_	PES	MBR	Phase inversion	Improved the hydrophilicity	[329]
Ag	PVP	UF	Phase inversion	Antimicrobial functionality	[330]
Ag-Cu_2_O	PSF	UF	Phase inversion	Enhanced antibacterial properties	[331]
Ag	PU	UF	Electrospinning	Antimicrobial functionality	[332]
Clay	PVDF	UF	Phase inversion	Mechanical property; Hydrophilicity	[333]

**Table 7:** List of different studies on dynamic membranes with different support materials.

Support Material	Application Type	Treatment	Reference
mesh	MBR	Municipal wastewater treatment	[353]
woven fabric	MBR	Wastewater treatment	[356]
non-woven fabric	UF	Degradation of 4-chlorophenol	[357]
ceramic tube	MF	Oily wastewater treatment	[358]
ceramic membranes	UF	Oil-water emulsion separation	[350]
stainless steel tube	UF	Concentration of protein hemoglobin	[359]
reverse osmosis (RO) membrane	RO	Concentration/purification of Co(II) ion	[360]
MF membrane	MF	Rejection of macromolecules	[361]
UF membrane	RO	Concentration/purification of Co(II) ion	[360]
Porous carbon	MF	Oil-water emulsion separation	[362]

**Table 8:** List of different studies on different dynamic material layers for water treatment applications.

Dynamic Material	Support Material	Application Type	Treatment	Flux	Removal Efficiency	Reference
ZrOCl	Ceramic	UF	Glucose removal	16–34 LMH	1.6–5.8 (glucose removal)	[363]
Fe_2_O_3_	Ceramic	UF	Oil-water emulsion separation		97.8 (COD removal)	[350]
Kaolin clay	Ceramic	MF	Oil removal	400 LMH	96% (oil removal)	[351]
MnO_2_	Ceramic	UF	Oil removal	1000 LMH	-	[358]
TiO_2_	Porous carbon	MF	Oil-water emulsion separation	240 LMH	98% (oil removal)	[362]
Diatomite	Stainless steel mesh	MBR	Surface water treatment	92 LMH	95% (turbidity)	[364]
CaCO_3_	non-woven fabric	PMR	Treatment of halogenated compounds in water	-	72% (removal of 2,4,6-Tribromophenol). And 75% (removal of 2,4-Dichlorophenol)	[365]
Kaolin–MnO_2_	Al_2_O_3_ porous ceramic	MF	Oil removal	120.1–153.2 LMH	98.2–99.9 (Oil removal)	[358]
Mg(OH)_2_	Al_2_O_3_ ceramic tubes	MF	Wastewater treatment	1 LMH	98 (TOC removal)	[366]
